# Supplementary material for: Early heart rate variability changes during acute fetal inflammatory response syndrome: An experimental study in a fetal sheep model
Source: PLoS One. 2023 Nov 30;18(11):e0293926. doi: 10.1371/journal.pone.0293926 (PMC10688759; doi:10.1371/journal.pone.0293926)
Supplement: Supplementary file 1 [file pone.0293926.s001.odt]

S1 Table. Hemodynamic, blood gas and biochemical measures in LPS and control groups

| sheep number | MAP (mmhg) | pH | PCO2 mmHg | PO2 mmHg | BE | HCO3 mmHg | Lactates mmol/L | Il6 pg/ml |
| --- | --- | --- | --- | --- | --- | --- | --- | --- |
| 15323 | 45 | 7,4 | 43 | 17 | 2 | 26 | 2,41 | 7,8 |
| 15323 | 48 | 7,37 | 38,2 | 22 | -3 | 21,8 | 2,2 | 50,28 |
| 15323 | 44 | 7,38 | 44 | 21 | 2 | 26,3 | 2,89 | 611,478 |
| 15323 | 42 | 7,37 | 47,9 | 19 | 2 | 27,3 | 2,86 | 1749,87 |
| 15323 | 37 | 7,37 | 49,2 | 20 | 3 | 28,2 | 2,94 | 1801,52 |
| 15323 | 37 | 7,39 | 45,5 | 20 | 3 | 27,1 | 2,64 | 1352,4 |
| 15323 | 36 | 7,39 | 43,7 | 16 | 1 | 26 | 2,24 | 1312,35 |
| 32093 | 43 | 7,38 | 47,3 | 16 | 3 | 27,8 | 2,13 | 7,8 |
| 32093 | 48 | 7,36 | 48,3 | 18 | 2 | 27,1 | 2,02 | 78,749 |
| 32093 | 42 | 7,28 | 53,1 | 16 | -1 | 24,9 | 2,43 | 1446,28 |
| 32093 | 43 | 7,26 | 57,6 | 12 | -1 | 25,7 | 3,89 | 4674,58 |
| 32093 | 41 | 7,27 | 55,3 | 14 | -1 | 25,2 | 4,4 | 4722,9 |
| 32093 | 40 | 7,27 | 52,5 | 13 | -2 | 24 | 4,88 | 3915,25 |
| 32093 | 40 | 7,3 | 51,2 | 15 | -1 | 24,8 | 5,45 | 3764,91 |
| 32182 | 42 | 7,41 | 42,9 | 22 | 3 | 27,1 | 2,26 | 7,8 |
| 32182 | 45 | 7,39 | 43,6 | 20 | 2 | 26,2 | 2,57 | 79,791 |
| 32182 | 46 | 7,34 | 49,3 | 18 | 1 | 26,1 | 3,79 | 1224,82 |
| 32182 | 45 | 7,33 | 46,6 | 19 | -1 | 24,1 | 4,52 | 3166,23 |
| 32182 | 46 | 7,36 | 46,1 | 17 | 1 | 25,8 | 4,49 | 3327,29 |
| 32182 | 41 | 7,36 | 47,7 | 18 | 2 | 26,2 | 4,75 | 3651,3 |
| 32182 | 47 | 7,35 | 49,7 | 18 | 2 | 27 | 4,86 | 3181,63 |
| 40109 | 48 | 7,39 | 46,7 | 15 | 3 | 27,6 | 3,22 | 9,24 |
| 40109 | 50 | 7,36 | 50,3 | 13 | 3 | 28,1 | 3,38 | 54,11 |
| 40109 | 46 | 7,3 | 52,3 | 15 | -1 | 25,3 | 5,27 | 1472,17 |
| 40109 | 43 | 7,27 | 56,6 | 13 | 0 | 25,9 | 7,11 | 3740,58 |
| 40109 | 46 | 7,28 | 50,6 | 13 | -3 | 23,3 | 7,31 | 3333,65 |
| 40109 | 41 | 7,27 | 55,6 | 12 | -1 | 25 | 9,46 | 4198,03 |
| 40109 | 41 | 7,25 | 57,2 | 15 | -2 | 24,6 | 10,52 | 3498,91 |
| 93961 | 44 | 7,41 | 51 | 15 | 7 | 31,7 | 1,99 | 7,8 |
| 93961 | 47 | 7,39 | 50,7 | 20 | 6 | 30,4 | 2,35 | 12,045 |
| 93961 | 50 | 7,39 | 53,7 | 20 | 5 | 29,8 | 2,78 | 899,374 |
| 93961 | 47 | 7,34 | 58,3 | 16 | 6 | 30,9 | 3,57 | 2450,06 |
| 93961 | 52 | 7,33 | 54 | 15 | 3 | 28 | 3,82 | 2327,5 |
| 93961 | 44 | 7,34 | 56,6 | 14 | 5 | 30,3 | 5,44 | 2102,52 |
| 93961 | 41 | 7,34 | 56,8 | 15 | 5 | 30,3 | 5,86 | 1989,69 |
| 93973 | 50 | 7,39 | 54,4 | 17 | 8 | 32,6 | 1,94 | 7,8 |
| 93973 | 51 | 7,39 | 55 | 16 | 8 | 32,5 | 2,78 | 15,618 |
| 93973 | 49 | 7,4 | 51,7 | 19 | 7 | 31,3 | 3,31 | 611,478 |
| 93973 | 51 | 7,38 | 54,4 | 16 | 7 | 31,5 | 4,37 | 2100,07 |
| 93973 | 46 | 7,36 | 59 | 14 | 8 | 32,9 | 4,68 | 1972,93 |
| 93973 | 42 | 7,36 | 58,5 | 16 | 7 | 32,2 | 5,14 | 2007,71 |
| 93973 | 40 | 7,37 | 57,2 | 16 | 7 | 32,3 | 4,89 | 1912,37 |
| 83782 | 57 | 7,38 | 48 | 22 | 4 | 28,1 | 2,77 | 7,8 |
| 83782 | 58 | 7,39 | 47,7 | 21 | 4 | 28,4 | 2,34 | 94,936 |
| 83782 | 50 | 7,38 | 48,1 | 20 | 3 | 27,7 | 3,01 | 198,433 |
| 83782 | 47 | 7,32 | 52,5 | 16 | 1 | 26,7 | 4,66 | 3127,95 |
| 83782 | 45 | 7,33 | 49,2 | 18 | 0 | 25,3 | 6,13 | 3663,22 |
| 83782 | 49 | 7,32 | 51,4 | 17 | 1 | 26,1 | 7,22 | 4219,00 |
| 83782 | 41 | 7,33 | 50,1 | 17 | 0 | 25,2 | 8,24 | 3911,65 |
| 83813 | 46 | 7,39 | 48,5 | 21 | 5 | 29 | 2 | 7,8 |
| 83813 | 47 | 7,38 | 51,6 | 23 | 5 | 29,8 | 2,41 | 27,962 |
| 83813 | 43 | 7,33 | 51,7 | 23 | 1 | 26,9 | 4,06 | 1410,89 |
| 83813 | 42 | 7,31 | 54,4 | 20 | 1 | 27 | 5,78 | 6317,99 |
| 83813 | 47 | 7,3 | 54,1 | 18 | 0 | 26,1 | 6,98 | 6102,7 |
| 83813 | 49 | 7,3 | 52,3 | 17 | -1 | 25,2 | 8,09 | 6144,76 |
| 83813 | 46 | 7,31 | 52,5 | 19 | 0 | 26,1 | 8,98 | 5863,53 |
| 83685 | 60 | 7,37 | 54 | 19 | 6 | 30,8 | 2,66 | 7,8 |
| 83685 | 60 | 7,38 | 52,7 | 19 | 6 | 30,6 | 2,53 | 7,8 |
| 83685 | 58 | 7,39 | 52,6 | 20 | 7 | 31,1 | 2,35 | 7,8 |
| 83685 | 57 | 7,4 | 52,3 | 19 | 7 | 31,5 | 2,32 | 7,8 |
| 83685 | 51 | 7,38 | 52,9 | 14 | 6 | 30,6 | 2,12 | 7,8 |
| 83685 | 65 | 7,38 | 52,1 | 20 | 6 | 30,4 | 2,27 | 7,8 |
| 83685 | 67 | 7,37 | 55,5 | 15 | 7 | 31,8 | 2,4 | 7,8 |
| 83720 | 39 | 7,42 | 43,2 | 24 | 3 | 27,3 | 2,55 | 7,8 |
| 83720 | 42 | 7,43 | 38,2 | 20 | 3 | 27,2 | 2,66 | 7,8 |
| 83720 | 41 | 7,41 | 45,7 | 22 | 4 | 28,2 | 2,68 | 7,8 |
| 83720 | 43 | 7,4 | 38,2 | 22 | -1 | 23,3 | 2,12 | 7,8 |
| 83720 | 43 | 7,41 | 45,1 | 21 | 4 | 28,1 | 2,41 | 7,8 |
| 83720 | 40 | 7,4 | 46,3 | 18 | 4 | 28,4 | 2,37 | 7,8 |
| 83720 | 40 | 7,4 | 47,9 | 21 | 4 | 28,8 | 2,37 | 7,8 |
| 83733 | 65 | 7,45 | 46,3 | 23 | 8 | 31,8 | 2,51 | 7,8 |
| 83733 | 64 | 7,45 | 44,1 | 27 | 7 | 30,1 | 2,43 | 7,8 |
| 83733 | 59 | 7,43 | 44,1 | 27 | 5 | 28,9 | 2,39 | 7,8 |
| 83733 | 64 | 7,44 | 45,1 | 26 | 6 | 30 | 2 | 7,8 |
| 83733 | 66 | 7,43 | 47,3 | 25 | 7 | 30,7 | 1,93 | 7,8 |
| 83733 | 64 | 7,43 | 48,3 | 25 | 7 | 31,1 | 1,96 | 7,8 |
| 83733 | 63 | 7,43 | 47,1 | 28 | 7 | 30,8 | 1,97 | 7,8 |
| 83733 | 57 | 7,44 | 48,8 | 26 | 8 | 32,1 | 2,07 | 7,8 |
| 83733 | 51 | 7,4 | 46,2 | 26 | 4 | 28,3 | 1,75 | 7,8 |
| 40293 | 45 | 7,39 | 50,3 | 16 | 6 | 30,1 | 2,85 | 7,8 |
| 40293 | 47 | 7,39 | 51,4 | 15 | 6 | 30,2 | 2,79 | 7,8 |
| 40293 | 43 | 7,38 | 52 | 18 | 6 | 30,4 | 2,83 | 7,8 |
| 40293 | 41 | 7,4 | 48,7 | 19 | 6 | 29,8 | 2,77 | 7,8 |
| 40293 | 42 | 7,41 | 47,5 | 17 | 5 | 29,4 | 2,69 | 7,8 |
| 40293 | 41 | 7,39 | 50,8 | 17 | 6 | 30,1 | 2,55 | 7,8 |
| 40293 | 43 | 7,38 | 53,3 | 16 | 6 | 30,6 | 2,21 | 7,8 |
| 16543 | 48 | 7,42 | 48,1 | 17 | 7 | 30,9 | 2,8 | 7,8 |
| 16543 | 45 | 7,43 | 47 | 19 | 7 | 31 | 2,38 | 7,8 |
| 16543 | 45 | 7,43 | 49,6 | 17 | 8 | 32,2 | 2,08 | 7,8 |
| 16543 | 47 | 7,43 | 50,5 | 16 | 9 | 32,5 | 1,95 | 7,8 |
| 16543 | 48 | 7,43 | 49,2 | 16 | 8 | 32,1 | 2,13 | 7,8 |
| 16543 | 54 | 7,44 | 47,6 | 17 | 8 | 31,7 | 2,1 | 7,8 |
| 16543 | 48 | 7,42 | 45 | 16 | 5 | 28,5 | 2,09 | 7,8 |
| 83452 | 41 | 7,39 | 50,4 | 16 | 6 | 30,2 | 2,42 | 7,8 |
| 83452 | 42 | 7,41 | 48 | 18 | 6 | 30,1 | 1,9 | 7,8 |
| 83452 | 45 | 7,41 | 48,3 | 18 | 6 | 30,2 | 1,87 | 7,8 |
| 83452 | 43 | 7,41 | 47,8 | 17 | 6 | 29,8 | 1,76 | 7,8 |
| 83452 | 41 | 7,39 | 49,8 | 19 | 5 | 29,8 | 1,62 | 7,8 |
| 83452 | 38 | 7,4 | 49,5 | 19 | 6 | 30 | 1,65 | 7,8 |
| 83452 | 43 | 7,4 | 47,8 | 18 | 5 | 29 | 1,77 | 7,8 |
| 94042 | 51 | 7,41 | 44,6 | 20 | 4 | 28 | 1,99 | 7,8 |
| 94042 | 52 | 7,41 | 49,4 | 16 | 6 | 30,3 | 1,8 | 7,8 |
| 94042 | 48 | 7,402 | 45,7 | 19 | 4 | 27,9 | 1,89 | 7,8 |
| 94042 | 41 | 7,4 | 46,5 | 20 | 4 | 28,3 | 1,86 | 7,8 |
| 94042 | 49 | 7,39 | 48,1 | 18 | 5 | 29,2 | 1,65 | 7,8 |
| 94042 | 43 | 7,4 | 44,9 | 21 | 3 | 27,2 | 1,57 | 7,8 |
| 94042 | 47 | 7,39 | 47,4 | 18 | 3 | 27,9 | 1,64 | 7,8 |

S1 Table. Hemodynamic, blood gas and biochemical measures in LPS and control groups

MAP = mean arterial pressure, IL6 = interleukin 6
