# Supplementary material for: Early heart rate variability changes during acute fetal inflammatory response syndrome: An experimental study in a fetal sheep model
Source: PLoS One. 2023 Nov 30;18(11):e0293926. doi: 10.1371/journal.pone.0293926 (PMC10688759; doi:10.1371/journal.pone.0293926)
Supplement: Supplementary file 2 [file pone.0293926.s002.odt]

| sheep number | LPS | Time | SDNN | RMSSD | HR | alpha1 | alpha2 | apen | sd1 | sd2 | FSI | LF | HF | LFHF | LTV | STV |
| --- | --- | --- | --- | --- | --- | --- | --- | --- | --- | --- | --- | --- | --- | --- | --- | --- |
| 15323 | 1 | H0 | 7,31 | 7,62 | 171,98 | 6,72E-17 | 6,9E-18 | 0,6185 | 0,005 | 0,0086 | 53,17 | 1,70 | 6,50 | 0,24 | 40,12 | 3,35 |
| 15323 | 1 | H1 | 7,80 | 7,57 | 180,39 | 8,81E-17 | 8,44E-18 | 0,6579 | 0,005 | 0,009 | 44,91 | 1,72 | 5,51 | 0,26 | 29,06 | 2,45 |
| 15323 | 1 | H2 | 8,92 | 6,48 | 165,94 | 5,42E-17 | 3,89E-18 | 0,6411 | 0,005 | 0,0112 | 40,24 | 1,74 | 4,60 | 0,37 | 33,97 | 2,40 |
| 15323 | 1 | H3 | 10,45 | 9,00 | 169,82 | 4,12E-17 | 2,14E-18 | 0,562 | 0,006 | 0,0127 | 42,45 | 1,57 | 5,14 | 0,36 | 19,37 | 1,75 |
| 15323 | 1 | H4 | 9,89 | 10,08 | 187,91 | 5,25E-17 | 3,14E-18 | 0,5326 | 0,007 | 0,0114 | 44,26 | 1,53 | 5,59 | 0,29 | 55,73 | 4,25 |
| 15323 | 1 | H5 | 8,84 | 10,09 | 197,31 | 9,28E-17 | 5,48E-18 | 0,5906 | 0,007 | 0,0096 | 52,14 | 1,58 | 7,19 | 0,27 | 34,56 | 3,09 |
| 15323 | 1 | H6 | 8,69 | 9,84 | 190,08 | 8,33E-17 | 9,05E-18 | 0,6001 | 0,007 | 0,0094 | 54,90 | 1,45 | 7,56 | 0,23 | 32,34 | 2,81 |
| 32093 | 1 | H0 | 10,05 | 11,59 | 171,58 | 8,85E-17 | 5,92E-18 | 0,6628 | 0,008 | 0,0111 | 55,28 | 1,50 | 8,32 | 0,13 | 25,36 | 2,27 |
| 32093 | 1 | H1 | 9,96 | 8,48 | 144,33 | 3,99E-17 | 2,19E-18 | 0,5748 | 0,006 | 0,0124 | 52,01 | 1,66 | 5,95 | 0,32 | 30,50 | 2,85 |
| 32093 | 1 | H2 | 19,34 | 15,53 | 165,38 | -1,9E-17 | -1,7E-18 | 0,4894 | 0,011 | 0,0244 | 51,89 | 1,73 | 5,58 | 0,30 | 73,79 | 6,66 |
| 32093 | 1 | H3 | 16,36 | 12,47 | 154,93 | 2,9E-18 | -9,5E-19 | 0,5278 | 0,009 | 0,0209 | 57,73 | 1,68 | 6,11 | 0,33 | 56,63 | 5,44 |
| 32093 | 1 | H4 | 19,82 | 12,85 | 163,57 | -1,3E-17 | -1,1E-18 | 0,5143 | 0,009 | 0,026 | 43,63 | 1,98 | 5,05 | 0,46 | 63,79 | 6,07 |
| 32093 | 1 | H5 | 21,28 | 17,89 | 181,12 | -3,5E-17 | -2,3E-18 | 0,4934 | 0,013 | 0,0265 | 55,85 | 1,65 | 6,79 | 0,26 | 57,61 | 6,13 |
| 32093 | 1 | H6 | 13,25 | 10,73 | 202,65 | 3,19E-18 | 3,6E-19 | 0,4473 | 0,008 | 0,0167 | 49,06 | 1,83 | 5,61 | 0,42 | 43,75 | 4,68 |
| 32182 | 1 | H0 | 13,73 | 16,64 | 175,04 | 6,09E-17 | 6,04E-18 | 0,3859 | 0,012 | 0,0149 | 67,06 | 1,68 | 8,71 | 0,20 | 33,54 | 3,53 |
| 32182 | 1 | H1 | 12,16 | 15,81 | 187,38 | 6,73E-17 | 5,06E-18 | 0,3819 | 0,011 | 0,0124 | 60,61 | 1,78 | 8,75 | 0,27 | 34,52 | 3,50 |
| 32182 | 1 | H2 | 16,64 | 10,81 | 174,58 | -1,1E-17 | -1,8E-18 | 0,5017 | 0,008 | 0,0216 | 44,43 | 1,74 | 5,03 | 0,33 | 59,85 | 5,52 |
| 32182 | 1 | H3 | 24,70 | 14,20 | 151,26 | -4,7E-17 | -3,3E-18 | 0,4716 | 0,01 | 0,0327 | 44,19 | 1,83 | 4,25 | 0,37 | 79,42 | 7,96 |
| 32182 | 1 | H4 | 11,28 | 8,77 | 189,11 | 2,81E-17 | 2,02E-18 | 0,552 | 0,006 | 0,0141 | 44,45 | 2,00 | 5,32 | 0,36 | 43,13 | 4,38 |
| 32182 | 1 | H5 | 10,62 | 8,15 | 206,89 | 3,73E-17 | 4,02E-18 | 0,5959 | 0,006 | 0,0133 | 43,57 | 1,82 | 5,71 | 0,49 | 37,37 | 3,34 |
| 32182 | 1 | H6 | 9,12 | 7,95 | 218,66 | 4,56E-17 | 2,81E-18 | 0,5828 | 0,006 | 0,0111 | 46,79 | 1,86 | 6,08 | 0,31 | 35,23 | 3,19 |
| 40109 | 1 | H0 | 6,29 | 4,11 | 168,49 | 8,27E-17 | 4,32E-18 | 0,6762 | 0,003 | 0,0082 | 42,72 | 2,02 | 4,35 | 0,54 | 36,01 | 3,10 |
| 40109 | 1 | H1 | 12,89 | 6,90 | 171,35 | 9,95E-18 | -1,2E-19 | 0,5272 | 0,005 | 0,0172 | 43,69 | 1,87 | 4,04 | 0,42 | 47,38 | 4,62 |
| 40109 | 1 | H2 | 11,00 | 5,31 | 172,94 | 1,79E-17 | 9,84E-19 | 0,5949 | 0,004 | 0,0148 | 42,18 | 1,83 | 4,00 | 0,64 | 42,60 | 3,76 |
| 40109 | 1 | H3 | 13,18 | 5,88 | 155,31 | 5,7E-18 | -6,2E-19 | 0,5521 | 0,004 | 0,0178 | 42,51 | 2,01 | 3,49 | 0,49 | 52,36 | 5,23 |
| 40109 | 1 | H4 | 14,23 | 5,48 | 165,16 | 1,05E-17 | 6,73E-19 | 0,5444 | 0,004 | 0,0194 | 43,78 | 2,06 | 3,73 | 0,71 | 54,97 | 5,38 |
| 40109 | 1 | H5 | 14,70 | 13,50 | 183,50 | 5,22E-17 | 3,79E-18 | 0,598 | 0,01 | 0,0172 | 48,83 | 2,06 | 5,19 | 0,32 | 29,48 | 2,96 |
| 40109 | 1 | H6 | 9,12 | 5,55 | 187,39 | 4,7E-17 | 4,68E-18 | 0,589 | 0,004 | 0,012 | 46,54 | 1,93 | 4,93 | 0,51 | 41,99 | 4,07 |
| 93961 | 1 | H0 | 10,20 | 6,79 | 189,11 | 3,7E-17 | 2,52E-18 | 0,5571 | 0,005 | 0,0133 | 52,96 | 2,14 | 5,19 | 0,39 | 42,60 | 4,94 |
| 93961 | 1 | H1 | 7,22 | 5,26 | 156,40 | 7,8E-17 | 6,09E-18 | 0,617 | 0,004 | 0,0093 | 45,31 | 1,92 | 5,12 | 0,36 | 37,91 | 3,55 |
| 93961 | 1 | H2 | 17,69 | 10,56 | 194,97 | -1,8E-17 | -2,2E-18 | 0,4716 | 0,007 | 0,0234 | 47,01 | 1,81 | 4,68 | 0,44 | 73,74 | 7,05 |
| 93961 | 1 | H3 | 21,52 | 15,02 | 163,25 | -2,6E-17 | -1,8E-18 | 0,453 | 0,011 | 0,028 | 50,82 | 2,00 | 5,13 | 0,55 | 70,98 | 7,44 |
| 93961 | 1 | H4 | 16,53 | 11,62 | 216,21 | -9,6E-18 | -1,7E-19 | 0,4448 | 0,008 | 0,0215 | 48,29 | 1,83 | 5,36 | 0,32 | 65,12 | 6,00 |
| 93961 | 1 | H5 | 19,14 | 13,55 | 191,79 | -2E-17 | -2,1E-18 | 0,3796 | 0,01 | 0,0248 | 53,64 | 1,83 | 5,60 | 0,38 | 64,72 | 6,94 |
| 93961 | 1 | H6 | 18,70 | 17,35 | 186,09 | -6E-18 | 4,43E-19 | 0,3084 | 0,012 | 0,0228 | 57,63 | 1,56 | 6,74 | 0,20 | 60,11 | 6,23 |
| 93973 | 1 | H0 | 5,59 | 3,61 | 200,38 | 1,07E-16 | 1,13E-17 | 0,6091 | 0,003 | 0,0071 | 45,54 | 1,80 | 4,80 | 0,53 | 24,12 | 2,39 |
| 93973 | 1 | H1 | 6,93 | 4,00 | 177,60 | 7,07E-17 | 4,52E-18 | 0,6532 | 0,003 | 0,0092 | 47,58 | 2,01 | 4,53 | 0,42 | 25,16 | 2,58 |
| 93973 | 1 | H2 | 6,68 | 5,05 | 182,77 | 7,1E-17 | 4,86E-18 | 0,6236 | 0,004 | 0,0085 | 45,21 | 1,80 | 5,00 | 0,42 | 25,98 | 2,63 |
| 93973 | 1 | H3 | 10,07 | 7,25 | 179,90 | 3,71E-17 | 1,9E-18 | 0,5805 | 0,005 | 0,0129 | 50,77 | 1,87 | 5,08 | 0,35 | 36,57 | 3,56 |
| 93973 | 1 | H4 | 7,30 | 4,50 | 199,00 | 5,8E-17 | 2,74E-18 | 0,6241 | 0,003 | 0,0096 | 42,84 | 1,67 | 4,93 | 0,60 | 25,80 | 2,22 |
| 93973 | 1 | H5 | 3,33 | 3,17 | 216,66 | 1,85E-16 | 1,29E-17 | 0,5872 | 0,002 | 0,0038 | 47,65 | 1,73 | 5,72 | 0,29 | 23,03 | 1,74 |
| 93973 | 1 | H6 | 5,21 | 3,58 | 203,88 | 9,81E-17 | 5,86E-18 | 0,6111 | 0,003 | 0,0067 | 39,43 | 1,79 | 4,51 | 0,51 | 21,80 | 1,87 |
| 83782 | 1 | H0 | 3,92 | 3,50 | 166,57 | 1,36E-16 | 1,14E-17 | 0,6233 | 0,002 | 0,0048 | 51,41 | 1,65 | 6,13 | 0,40 | 12,37 | 0,82 |
| 83782 | 1 | H1 | 5,17 | 4,00 | 160,24 | 1,01E-16 | 7,25E-18 | 0,6475 | 0,003 | 0,0065 | 45,86 | 1,80 | 4,99 | 0,65 | 76,71 | 5,48 |
| 83782 | 1 | H2 | 8,04 | 5,05 | 173,83 | 5,29E-17 | 4,45E-18 | 0,639 | 0,004 | 0,0105 | 45,27 | 1,73 | 5,19 | 0,47 | 33,37 | 3,24 |
| 83782 | 1 | H3 | 41,61 | 28,02 | 180,23 | -6,6E-17 | -6,1E-18 | 0,4075 | 0,02 | 0,0529 | 45,29 | 1,64 | 4,09 | 0,47 | 79,90 | 6,36 |
| 83782 | 1 | H4 | 16,85 | 7,69 | 175,19 | -8,8E-18 | -7,5E-19 | 0,5226 | 0,005 | 0,0227 | 39,65 | 1,84 | 3,76 | 0,69 | 69,25 | 4,67 |
| 83782 | 1 | H5 | 8,96 | 4,78 | 201,23 | 3,19E-17 | 2,52E-18 | 0,6281 | 0,003 | 0,0119 | 41,19 | 1,77 | 4,20 | 0,40 | 84,18 | 7,80 |
| 83782 | 1 | H6 | 7,87 | 4,73 | 248,26 | 8,76E-17 | 6,12E-18 | 0,6771 | 0,003 | 0,0102 | 36,68 | 1,85 | 4,52 | 0,42 | 79,95 | 6,86 |
| 83813 | 1 | H0 | 8,85 | 5,66 | 174,10 | 5,44E-17 | 3,49E-18 | 0,558 | 0,004 | 0,0116 | 49,52 | 2,01 | 4,68 | 0,40 | 28,63 | 2,94 |
| 83813 | 1 | H1 | 14,61 | 10,52 | 197,47 | 8,98E-18 | -2,7E-19 | 0,5312 | 0,007 | 0,0188 | 56,93 | 1,86 | 6,40 | 0,33 | 53,04 | 5,72 |
| 83813 | 1 | H2 | 19,31 | 15,37 | 172,82 | -2,7E-18 | 8,89E-19 | 0,4468 | 0,011 | 0,0242 | 65,80 | 1,80 | 6,54 | 0,31 | 56,12 | 6,36 |
| 83813 | 1 | H3 | 15,09 | 10,49 | 159,42 | 2,55E-17 | 2,41E-18 | 0,4584 | 0,007 | 0,0194 | 52,01 | 1,79 | 5,54 | 0,29 | 51,44 | 5,32 |
| 83813 | 1 | H4 | 19,23 | 12,52 | 188,56 | -2E-17 | -1,2E-18 | 0,4398 | 0,009 | 0,0252 | 52,14 | 2,10 | 5,21 | 0,34 | 61,37 | 0,70 |
| 83813 | 1 | H5 | 10,61 | 7,63 | 234,93 | 4,4E-17 | 3,85E-18 | 0,6042 | 0,005 | 0,0137 | 42,12 | 1,88 | 5,41 | 0,39 | 46,36 | 4,45 |
| 83813 | 1 | H6 | 12,96 | 8,47 | 239,80 | 9,89E-18 | 1,69E-19 | 0,486 | 0,006 | 0,017 | 52,49 | 2,05 | 5,91 | 0,37 | 40,85 | 4,81 |
| 83685 | 0 | H0 | 12,75 | 7,53 | 175,21 | 1,62E-17 | 2,38E-18 | 0,5508 | 0,005 | 0,0169 | 46,88 | 2,02 | 4,57 | 0,41 | 47,44 | 4,67 |
| 83685 | 0 | H1 | 12,31 | 7,27 | 178,37 | 9,81E-18 | 2,13E-19 | 0,5487 | 0,005 | 0,0163 | 59,55 | 1,64 | 5,35 | 0,42 | 47,13 | 4,50 |
| 83685 | 0 | H2 | 9,26 | 9,08 | 168,65 | 6,57E-17 | 5,83E-18 | 0,6111 | 0,006 | 0,011 | 53,24 | 1,60 | 6,03 | 0,11 | 25,87 | 2,38 |
| 83685 | 0 | H3 | 6,46 | 4,05 | 180,01 | 6,65E-17 | 3,31E-18 | 0,653 | 0,003 | 0,0084 | 38,13 | 1,72 | 3,96 | 0,38 | 27,12 | 2,36 |
| 83685 | 0 | H4 | 8,19 | 5,34 | 194,76 | 3,75E-17 | 4,43E-18 | 0,607 | 0,004 | 0,0107 | 55,40 | 1,74 | 5,85 | 0,40 | 34,02 | 3,65 |
| 83685 | 0 | H5 | 8,58 | 5,27 | 212,73 | 5,53E-17 | 4,33E-18 | 0,5441 | 0,004 | 0,0113 | 43,37 | 1,80 | 4,47 | 0,41 | 34,55 | 3,46 |
| 83685 | 0 | H6 | 15,54 | 17,10 | 173,03 | 4,88E-17 | 4,87E-18 | 0,5381 | 0,012 | 0,0179 | 75,20 | 1,38 | 8,93 | 0,24 | 31,52 | 3,39 |
| 83720 | 0 | H0 | 8,34 | 5,80 | 167,92 | 6,84E-17 | 1,4E-18 | 0,5791 | 0,004 | 0,0107 | 44,69 | 1,99 | 5,30 | 0,49 | 47,94 | 4,50 |
| 83720 | 0 | H1 | 12,08 | 9,72 | 167,63 | 3,39E-17 | 3,58E-18 | 0,5648 | 0,007 | 0,0151 | 47,23 | 1,92 | 5,58 | 0,39 | 38,75 | 3,61 |
| 83720 | 0 | H2 | 9,53 | 7,67 | 164,38 | 4,26E-17 | 3,25E-18 | 0,5737 | 0,005 | 0,0119 | 50,33 | 1,82 | 5,54 | 0,41 | 33,00 | 3,00 |
| 83720 | 0 | H3 | 14,17 | 12,23 | 164,23 | 2,99E-17 | 1,59E-18 | 0,5774 | 0,009 | 0,0176 | 53,08 | 1,78 | 6,10 | 0,28 | 48,81 | 5,01 |
| 83720 | 0 | H4 | 13,19 | 10,35 | 169,61 | 2,19E-17 | 3,47E-18 | 0,5153 | 0,007 | 0,0166 | 58,41 | 1,89 | 5,81 | 0,34 | 52,40 | 4,81 |
| 83720 | 0 | H5 | 13,94 | 12,50 | 172,45 | 2,15E-17 | 3,44E-18 | 0,5209 | 0,009 | 0,0172 | 47,60 | 2,09 | 6,17 | 0,32 | 51,84 | 4,99 |
| 83720 | 0 | H6 | 13,57 | 13,24 | 180,15 | 3,1E-17 | 2,6E-18 | 0,5247 | 0,009 | 0,0159 | 57,02 | 1,88 | 6,52 | 0,19 | 44,24 | 4,19 |
| 83733 | 0 | H0 | 12,04 | 18,15 | 174,91 | 1,05E-16 | 7,3E-18 | 0,57 | 0,013 | 0,0107 | 81,20 | 1,06 | 11,33 | 0,15 | 25,44 | 2,73 |
| 83733 | 0 | H1 | 14,19 | 20,31 | 171,71 | 7,34E-17 | 6,99E-18 | 0,5281 | 0,014 | 0,0132 | 71,32 | 1,33 | 9,73 | 0,08 | 29,59 | 2,82 |
| 83733 | 0 | H2 | 12,07 | 15,82 | 176,34 | 5,74E-17 | 4,82E-18 | 0,5221 | 0,011 | 0,0123 | 72,01 | 1,53 | 9,36 | 0,22 | 27,74 | 3,08 |
| 83733 | 0 | H3 | 15,72 | 22,34 | 176,06 | 4,44E-17 | 1,51E-18 | 0,5299 | 0,016 | 0,015 | 71,23 | 1,49 | 9,38 | 0,26 | 39,40 | 3,70 |
| 83733 | 0 | H4 | 18,61 | 26,30 | 182,70 | 4,73E-17 | 2,34E-18 | 0,4962 | 0,019 | 0,0178 | 74,83 | 1,44 | 9,43 | 0,20 | 41,21 | 4,63 |
| 83733 | 0 | H5 | 17,41 | 23,70 | 168,36 | 3,37E-17 | 2,35E-18 | 0,5072 | 0,017 | 0,0175 | 82,26 | 1,64 | 9,46 | 0,13 | 34,60 | 4,11 |
| 83733 | 0 | H6 | 17,15 | 22,04 | 181,24 | 2,92E-17 | 8,16E-19 | 0,5086 | 0,016 | 0,0178 | 78,42 | 1,48 | 8,96 | 0,19 | 45,99 | 4,28 |
| 83733 | 0 | H24 | 10,24 | 11,56 | 178,61 | 8,45E-17 | 5,02E-18 | 0,5764 | 0,008 | 0,0114 | 66,23 | 1,63 | 8,90 | 0,19 | 22,45 | 2,40 |
| 83733 | 0 | H48 | 15,05 | 19,75 | 169,34 | 5,15E-17 | 4,12E-18 | 0,5436 | 0,014 | 0,0152 | 67,27 | 1,72 | 8,13 | 0,10 | 27,28 | 3,14 |
| 40293 | 0 | H0 | 10,77 | 6,41 | 183,23 | 3,54E-17 | 2,62E-18 | 0,541 | 0,005 | 0,0142 | 53,27 | 1,87 | 5,70 | 0,54 | 30,94 | 2,84 |
| 40293 | 0 | H1 | 12,85 | 8,56 | 209,73 | 1,73E-17 | 1,13E-18 | 0,4863 | 0,006 | 0,0167 | 47,72 | 1,70 | 5,96 | 0,37 | 31,49 | 3,11 |
| 40293 | 0 | H2 | 8,50 | 6,03 | 186,20 | 6,48E-17 | 6,05E-18 | 0,5741 | 0,004 | 0,0109 | 41,23 | 1,80 | 5,36 | 0,46 | 34,41 | 3,24 |
| 40293 | 0 | H3 | 11,05 | 5,80 | 178,96 | 3,67E-17 | 4,28E-18 | 0,5723 | 0,004 | 0,0148 | 39,25 | 1,97 | 4,05 | 0,70 | 41,79 | 3,76 |
| 40293 | 0 | H4 | 11,62 | 9,45 | 176,69 | 3,63E-17 | 8,73E-19 | 0,5748 | 0,007 | 0,0144 | 49,47 | 1,57 | 5,97 | 0,25 | 35,67 | 3,28 |
| 40293 | 0 | H5 | 6,76 | 4,30 | 172,89 | 5,59E-17 | 3,17E-18 | 0,6145 | 0,003 | 0,0088 | 42,59 | 1,74 | 4,10 | 0,38 | 34,46 | 3,41 |
| 40293 | 0 | H6 | 10,85 | 6,73 | 188,96 | 3,41E-17 | 1,94E-18 | 0,5789 | 0,005 | 0,0141 | 50,11 | 1,76 | 5,90 | 0,15 | 40,08 | 3,89 |
| 16543 | 0 | H0 | 28,63 | 25,66 | 229,51 | -3,5E-17 | -2,5E-18 | 0,2928 | 0,018 | 0,0342 | 49,60 | 1,60 | 5,32 | 0,38 | 34,58 | 3,37 |
| 16543 | 0 | H1 | 8,41 | 5,42 | 206,65 | 4,94E-17 | 4,05E-18 | 0,559 | 0,004 | 0,0109 | 50,97 | 1,72 | 5,83 | 0,41 | 34,82 | 2,75 |
| 16543 | 0 | H2 | 8,88 | 5,24 | 188,79 | 4,02E-17 | 6,2E-19 | 0,5603 | 0,004 | 0,0116 | 35,85 | 1,83 | 3,83 | 0,65 | 52,77 | 5,09 |
| 16543 | 0 | H3 | 11,63 | 9,13 | 175,17 | 6,06E-17 | 3,45E-18 | 0,5643 | 0,006 | 0,0145 | 44,10 | 1,71 | 6,53 | 0,29 | 41,25 | 3,26 |
| 16543 | 0 | H4 | 10,94 | 6,25 | 186,84 | 9,37E-18 | -1,9E-20 | 0,5566 | 0,004 | 0,0145 | 46,50 | 1,89 | 4,59 | 0,47 | 46,75 | 4,82 |
| 16543 | 0 | H5 | 9,39 | 7,33 | 177,04 | 5,14E-17 | 5,05E-18 | 0,589 | 0,005 | 0,0119 | 51,45 | 1,84 | 6,17 | 0,36 | 43,31 | 4,07 |
| 16543 | 0 | H6 | 29,22 | 25,27 | 230,29 | -4,4E-17 | -2,1E-18 | 0,3152 | 0,018 | 0,0356 | 45,10 | 1,70 | 5,47 | 0,23 | 43,20 | 3,71 |
| 83452 | 0 | H0 | 5,17 | 4,64 | 175,52 | 1,19E-16 | 7,55E-18 | 0,627 | 0,003 | 0,0062 | 53,40 | 1,57 | 7,15 | 0,14 | 28,25 | 3,19 |
| 83452 | 0 | H1 | 6,78 | 6,09 | 203,87 | 1,03E-16 | 6,75E-18 | 0,5641 | 0,004 | 0,0083 | 48,30 | 1,71 | 7,63 | 0,32 | 22,52 | 2,16 |
| 83452 | 0 | H2 | 8,00 | 6,44 | 178,73 | 6,92E-17 | 4,99E-18 | 0,5765 | 0,005 | 0,0101 | 49,32 | 2,12 | 6,12 | 0,36 | 24,76 | 2,50 |
| 83452 | 0 | H3 | 8,37 | 8,74 | 169,26 | 7,74E-17 | 6,15E-18 | 0,588 | 0,006 | 0,0097 | 58,29 | 1,64 | 7,52 | 0,34 | 32,13 | 2,82 |
| 83452 | 0 | H4 | 9,73 | 8,53 | 173,44 | 6,3E-17 | 3,9E-18 | 0,5607 | 0,006 | 0,0119 | 54,22 | 2,00 | 6,74 | 0,41 | 29,37 | 3,25 |
| 83452 | 0 | H5 | 10,17 | 7,92 | 181,25 | 4,31E-17 | 3,37E-18 | 0,5643 | 0,006 | 0,0128 | 48,27 | 1,65 | 6,19 | 0,29 | 35,89 | 3,42 |
| 83452 | 0 | H6 | 6,54 | 4,94 | 187,04 | 7,91E-17 | 7,05E-18 | 0,5918 | 0,003 | 0,0084 | 46,04 | 2,02 | 6,01 | 0,41 | 28,15 | 2,71 |
| 94042 | 0 | H0 | 15,06 | 6,26 | 171,32 | -9,1E-18 | -9,5E-19 | 0,4931 | 0,004 | 0,0205 | 39,78 | 2,00 | 3,25 | 0,64 | 54,50 | 5,50 |
| 94042 | 0 | H1 | 15,39 | 6,36 | 164,30 | -1,2E-17 | 4,06E-19 | 0,5171 | 0,004 | 0,021 | 40,83 | 2,13 | 3,22 | 0,64 | 61,32 | 5,97 |
| 94042 | 0 | H2 | 9,50 | 5,21 | 178,39 | 3,23E-17 | 3,24E-18 | 0,6184 | 0,004 | 0,0127 | 39,13 | 2,00 | 3,75 | 0,50 | 51,14 | 4,47 |
| 94042 | 0 | H3 | 12,16 | 5,55 | 177,52 | 7,75E-18 | 1,17E-18 | 0,53 | 0,004 | 0,0164 | 40,11 | 2,05 | 3,47 | 0,58 | 49,76 | 4,24 |
| 94042 | 0 | H4 | 9,81 | 4,71 | 184,44 | 3,69E-17 | 1,19E-18 | 0,5417 | 0,003 | 0,0132 | 36,91 | 2,13 | 3,26 | 0,78 | 49,86 | 5,09 |
| 94042 | 0 | H5 | 13,12 | 6,96 | 213,81 | 1,73E-17 | 1,19E-18 | 0,5008 | 0,005 | 0,0175 | 38,85 | 1,79 | 3,57 | 0,47 | 74,70 | 7,50 |
| 94042 | 0 | H6 | 11,46 | 7,50 | 175,46 | 2,11E-17 | 7,8E-19 | 0,5323 | 0,005 | 0,015 | 44,29 | 2,01 | 4,26 | 0,39 | 48,73 | 5,06 |

S2 Tab. Heart rate variability measures in LPS and control groups

SDNN = standard deviation of normal to normal, R-R intervals, RMSSD = root mean square of successive differences, DFA = detrended fluctuation analysis, SD1 = Standard Deviation 1, SD2 = Standard Deviation 2, FSI = fetal stress index, ApEn = Approximate Entropy,

STV = short-term variability, LTV = long-term variability, LF = low frequencies, HF = high frequencies
