# Supplementary material for: Early heart rate variability changes during acute fetal inflammatory response syndrome: An experimental study in a fetal sheep model
Source: PLoS One. 2023 Nov 30;18(11):e0293926. doi: 10.1371/journal.pone.0293926 (PMC10688759; doi:10.1371/journal.pone.0293926)
Supplement: Attachment — Submitted filename: response to reviewers.docx [file pone.0293926.s003.docx]

**Geoffroy Chevalier**

Department of Obstetrics

Jeanne de Flandre Hospital, CHU Lille

Avenue Eugène Avinée, 59120 Loos, France

geoffroy.chevalier@chu-lille.fr

**Editorial Office**

“PLOS ONE

**Original article entitled**

“**Early heart rate variability changes during acute fetal inflammatory response syndrome: an experimental study in a fetal sheep model**”

Dear Editor,

We would like to thank you for giving us the opportunity to submit a corrected version of our work in your journal.

We also thank the reviewers for the time spent judging our manuscript and the quality of their comments. We have made every effort to respond to each of the comments and believe that the quality of the manuscript is greatly improved by the suggestions made to us.

Corrections appear in highlighted in the manuscript and are detailed in the table below.

We hope this revised article deserves publication in your prestigious journal,

Best regards,

On the behalf of the co-authors,

Doctor Geoffroy Chevalier, MD, PhD Student

**Journal requirements:**

| REQUIREMENTS | RESPONSES |
| --- | --- |
| 1. Please ensure that your manuscript meets PLOS ONE's style requirements, including those for file naming. | Following items have been corrected:  - Level 1 heading are in Bold type, 18pt  - Level 2 heading are in Bold type, 16pt  - References have been conformed to Plos one guidelines  - Authors and each affiliations have been corrected  - File naming has been corrected |
| 2. In your Data Availability statement, you have not specified where the minimal data set underlying the results described in your manuscript can be found. | We added Complete data about each fetus is provided in Supporting information. |
| 3. Please amend the manuscript submission data (via Edit Submission) to include author L. Storme | We include him via Edit Submission |

**Reviewer’s comments:**

| REVIEW | RESPONSE |
| --- | --- |
| **Reviewer 1:**  I request a fundamental change. You state that FSI, RMSSD, and HF correlate with parasympathetic activity, and that the lack of variation in these indices after LPS injection indicates no change in parasympathetic activity. Besides, you considers that both sympathetic and parasympathetic activity contribute to SDNN, and that the increase in SDNN in the absence of parasympathetic activation indicates that sympathetic activation has occurred. Regarding the fetal sheep HRV parameter, there is indeed substantial overlap in the sympathetic and parasympathetic spectrum, although HRV itself is predominantly ANS (PMID: 24014809).  It must be noted that each parameter does not strictly reflect direct sympathetic or parasympathetic individual changes (PMID: 25063795).  Also, the presence of tachycardia in H5 cannot be ruled out as a result of the production of catecholamines associated with parasympathetic activation. In fact, the timing of the tachycardia onset does not coincide with the SDNN elevation. How do you interpret this? It is also a significant leap of logic to say that significant changes in SDNN, DFA α1, DFA α2, SD2, LTV, and ApEn are detected prior to tachycardia and that these changes seem to be related to sympathetic activation and loss of signal complexity.  Why was there no change in LF/HF, which was shown to represent an imbalance between parasympathetic and sympathetic nerves? Consideration of this part of the question is lacking.  Lear CA et al. have shown that after repeated hypoxia, sympathetic control of FHRV is rapidly suppressed and parasympathetic control becomes essentially fully dominant (PMID: 32491938, 25864517, 32877241). While intermittent hypoxic exposure also elicits tachycardia, suggesting parasympathetic involvement, if there is clear evidence that a fetus exposed to inflammation becomes sympathetically dominant, it must be stated. If there is no evidence to back it up, then your discussion is a leap too far.  Also, as a fundamental issue, the general view from adult studies is that the relationship between ANS activity and spectral analysis of HRV is obscured at the extremes of physiology, and caution should be exercised in interpreting the results of HRV studies (PMID: 8093124). Fetal adaptation to inflammatory stress may be an extreme physiological situation that cannot be monitored in adults.  "Quantitation of any variability via whatever technique does not represent an analysis of the complex interactions underlying the variability, but merely a measurement of the resulting phenomenon."(PMID: 16645191), one must be careful in interpreting the results. | We thank you for the quality and the explanation of this comments.  We agreed that our results do not allow to clearly conclude to a sympathetic activation.  We change this part in our discussion and conclusion :  Discussion: l351-372  ~~FSI, RMSSD, and HF are known to be correlated with parasympathetic activity [20,23]. That we failed to find any fluctuations in these indices after LPS injection suggests an absence of change in parasympathetic activity. In contrast, after LPS injection, LTV, SD2, and SDNN increased from H2 to H4 in comparison to baseline. SD2 increased at H3 and LTV increased at H2 and H4 in comparison to control group. SD2 measures short and long-term HRV and is correlated with SDNN [21]. Both sympathetic and parasympathetic activities contribute to SDNN. Because SDNN increases in the absence of parasympathetic activation, we can presume that sympathetic activation occurred. The presence of tachycardia at H5 also supports the presence of sympathetic activation.~~  FSI, RMSSD, and HF has been associated with parasympathetic activity [20,23]. That we failed to find any fluctuations in these indices after LPS injection, could suggest an absence of change in parasympathetic activity. In contrast, after LPS injection, SDNN increased from H2 to H4 in comparison to baseline. Both sympathetic activity and parasympathetic activity contribute to SDNN. This increase of SDNN with no parasympathetic activation could suggest a sympathetic activation. However, a sympathetic activation with no parasympathetic activation should have increased the LF/HF ratio, what we did not highlight. Altogether, inhibition or activation of autonomic activities cannot be clearly established by our HRV indices changes.  It must be noted that each parameter does not strictly reflect direct sympathetic or parasympathetic individual changes [32] and there is a substantial overlap in the sympathetic and parasympathetic spectrum [33].  Conclusion:  Line 411-416:  ~~These changes appear related to sympathetic activation and loss of signal complexity~~  These changes appear to be mediated by an increase of global variability and a loss of signal complexity. |
| Please correct the statement "FSI reflects parasympathetic nervous system fluctuations" for the reasons discussed below. | Garabedian et al have found that FSI is correlated with parasympathetic activity. In fetal sheep, after intravenous  atropine, to inhibit parasympathetic tone, FSI was significantly decreased (1).  We have corrected the sentence: (l160)  ~~In previous experimental studies, we demonstrated that FSI reflects parasympathetic fluctuation and is correlated with acidosis [10,16,17,24,25].~~  In previous experimental studies, we demonstrated that FSI was correlated with acidosis and parasympathetic activation [10,16,17,17,24,25].  (1) Garabedian C, Champion C, Servan-  Schreiber E, Butruille L, Aubry E, Sharma D, et al.  (2017) A new analysis of heart rate variability in the  assessment of fetal parasympathetic activity: An  experimental study in a fetal sheep model. PLoS  ONE 12(7): e0180653. |
| Why did you not use Sample Entropy to evaluate the regularity and complexity of the time series? | To evaluate the regularity and complexity of the time series, we choose Approximate Entropy.  The advantage of Sample Entropy is its independence on time-series length; thus it can be computed from shorter HRV records. However, we had a large number of samples within the 64 s window used for HRV analysis (512 samples). This is why we have chosen to focus on Approximate Entropy |
| It is stated as Mann-Withney. Please correct the typo. | Typo has been corrected :  L182: were performed by Mann-Whitney test. |
| Fewer than half of the cases in the Control and LPS groups were singletons. It would be necessary to state whether there were twins or more. It is also necessary to mention whether there was any difference in response to LPS between twins and singletons, as has been the case in previous reports. | Only 3/8 fetus were singletons, 5/8 were twins in LPS group. Statistical analyses are not possible with only 3 fetuses.  Below, in this answer, we added a figure with the evolution of heart rate variability measures in LPS group for each fetus. We added complete data about each fetus in supporting information. Changes in singletons and twins seem similar.  We added in limits (l409): Third, due to small number of fetal sheep, it was not possible to research eventual differences in HRV modifications between singletons and twins. |
| Is the p-value of H2 for FSI a misstatement? | Yes, it was a misstatement  Correction: table 2: (FSI) p at H2:  ~~100~~ -> 1.00 |
| **Reviewer 2**  Line66-68: Authors describe “Unfortunately, current understanding of how infection/inflammation affects fetal HRV is limited, in part due to the heterogeneous nature of clinical infections [15].” While I, as an obstetrician and gynecologist, can very much understand what is being conveyed, I think it would be better to rephrase this statement to reflect that the diagnostic criteria for intrauterine infection or inflammation are not unified across pathology, biochemistry, and clinical judgment.  Also, the transition from this statement to lines 69-70 is abrupt. It should be described why a hypothesis was formulated that HRV analysis would be useful for early detection. | For better readability, we change these sentences : (l63-66)  ~~Unfortunately, current understanding of how infection/inflammation affects fetal HRV is limited, in part due to the heterogeneous nature of clinical infections [15].~~  ~~We hypothesized that HRV analysis can be used for early detection of acute FIRS.~~  “Unfortunately, current understanding of how infection/inflammation affects fetal HRV is limited [15].  Since HRV is correlated with SNA alterations and since it allows detection of neonatal inflammation, we hypothesized that HRV analysis can be used for early detection of acute FIRS”.  For better readability, we finally chose to not discuss the diversity of fetal infection and the variability of definition. In fact, our model focus on fetal inflammatory response syndrome, which is clearly defined and not on intrauterine infection and inflammation. |

LTV

|  | 15323 | 32093 | 32182 | 40109 | 93961 | 93373 | 83782 | 83813 |
| --- | --- | --- | --- | --- | --- | --- | --- | --- |
| H0 | 40.1171 | 25.3639 | 33.5387 | 36.0094 | 42.6002 | 24.1152 | 12.3747 | 28.6292 |
| H1 | 29.0551 | 30.5016 | 34.516 | 47.3849 | 37.9066 | 25.1564 | 76.7118 | 53.0435 |
| H2 | 33.9702 | 73.792 | 59.8474 | 42.5978 | 73.7419 | 25.979 | 33.365 | 56.1243 |
| H3 | 19.3697 | 56.6253 | 79.4155 | 52.363 | 70.9759 | 36.5677 | 79.9032 | 51.4446 |
| H4 | 55.7279 | 63.7929 | 43.1335 | 54.9703 | 65.1172 | 25.7998 | 69.2469 | 61.3669 |
| H5 | 34.5588 | 57.6122 | 37.3676 | 29.477 | 64.7202 | 23.0287 | 84.1751 | 46.3581 |
| H6 | 32.3352 | 43.7519 | 35.2253 | 41.9923 | 60.1085 | 21.8013 | 79.9503 | 40.8492 |

SD2

|  | 15323 | 32093 | 32182 | 40109 | 93961 | 93373 | 83782 | 83813 |
| --- | --- | --- | --- | --- | --- | --- | --- | --- |
| H0 | 0.00855899 | 0.0110939 | 0.0148925 | 0.00822208 | 0.0133479 | 0.00713092 | 0.00479221 | 0.0116093 |
| H1 | 0.00904063 | 0.0123663 | 0.0123742 | 0.0171983 | 0.00925169 | 0.00916198 | 0.00649062 | 0.0187948 |
| H2 | 0.0112398 | 0.0243877 | 0.0216059 | 0.0147871 | 0.0233564 | 0.00848325 | 0.010477 | 0.0241944 |
| H3 | 0.0127009 | 0.020927 | 0.032667 | 0.0178462 | 0.027991 | 0.0128964 | 0.0528699 | 0.0193801 |
| H4 | 0.0113634 | 0.026 | 0.0141018 | 0.0193828 | 0.0214772 | 0.00955861 | 0.0227074 | 0.0252378 |
| H5 | 0.00960536 | 0.026497 | 0.0132977 | 0.017233 | 0.0247523 | 0.00378182 | 0.0119082 | 0.0136519 |
| H6 | 0.00942257 | 0.0166929 | 0.0110736 | 0.0119543 | 0.0227858 | 0.00668872 | 0.0102238 | 0.0169889 |

ApEn

|  | 15323 | 32093 | 32182 | 40109 | 93961 | 93373 | 83782 | 83813 |
| --- | --- | --- | --- | --- | --- | --- | --- | --- |
| H0 | 0.618469 | 0.662793 | 0.385904 | 0.676245 | 0.557123 | 0.609122 | 0.623305 | 0.557973 |
| H1 | 0.657908 | 0.574774 | 0.381877 | 0.527192 | 0.61702 | 0.653176 | 0.647492 | 0.531157 |
| H2 | 0.641118 | 0.489351 | 0.501699 | 0.594939 | 0.4716 | 0.623606 | 0.639037 | 0.44677 |
| H3 | 0.561982 | 0.527767 | 0.471639 | 0.55209 | 0.453014 | 0.580519 | 0.407507 | 0.458374 |
| H4 | 0.53257 | 0.51432 | 0.551999 | 0.544417 | 0.44476 | 0.624147 | 0.522576 | 0.439845 |
| H5 | 0.590579 | 0.493445 | 0.595927 | 0.597963 | 0.379633 | 0.587209 | 0.628124 | 0.604222 |
| H6 | 0.600059 | 0.447311 | 0.582848 | 0.589031 | 0.308448 | 0.61109 | 0.677084 | 0.485961 |

FSI

|  | 15323 | 32093 | 32182 | 40109 | 93961 | 93373 | 83782 | 83813 |
| --- | --- | --- | --- | --- | --- | --- | --- | --- |
| H0 | 53.1709 | 55.2767 | 67.0619 | 42.7226 | 52.9581 | 45.5429 | 51.4077 | 49.521 |
| H1 | 44.9073 | 52.0096 | 60.6147 | 43.6922 | 45.3141 | 47.5846 | 45.8587 | 56.9286 |
| H2 | 40.2444 | 51.8865 | 44.4323 | 42.1772 | 47.0091 | 45.2082 | 45.269 | 65.7982 |
| H3 | 42.4529 | 57.7257 | 44.1854 | 42.5073 | 50.8241 | 50.7729 | 45.2936 | 52.0146 |
| H4 | 44.2627 | 43.6274 | 44.4507 | 43.7784 | 48.2892 | 42.8381 | 39.6497 | 52.1439 |
| H5 | 52.1413 | 55.8547 | 43.5653 | 48.8297 | 53.6391 | 47.6519 | 41.1869 | 42.1178 |
| H6 | 54.9004 | 49.0636 | 46.7926 | 46.5411 | 57.6259 | 39.4293 | 36.6768 | 52.4893 |

dfa A1

|  | 15323 | 32093 | 32182 | 40109 | 93961 | 93373 | 83782 | 83813 |
| --- | --- | --- | --- | --- | --- | --- | --- | --- |
| H0 | 6.71837e-17 | 8.85166e-17 | 6.0904e-17 | 8.26717e-17 | 3.69554e-17 | 1.07393e-16 | 1.36382e-16 | 5.44282e-17 |
| H1 | 8.81004e-17 | 3.98588e-17 | 6.72567e-17 | 9.95492e-18 | 7.79735e-17 | 7.07367e-17 | 1.00892e-16 | 8.98446e-18 |
| H2 | 5.42266e-17 | -1.9364e-17 | -1.08023e-17 | 1.79027e-17 | -1.79564e-17 | 7.10258e-17 | 5.29269e-17 | -2.69646e-18 |
| H3 | 4.11942e-17 | 2.89841e-18 | -4.69034e-17 | 5.6958e-18 | -2.59908e-17 | 3.7053e-17 | -6.55195e-17 | 2.54572e-17 |
| H4 | 5.24993e-17 | -1.27247e-17 | 2.80813e-17 | 1.05257e-17 | -9.58731e-18 | 5.79876e-17 | -8.76021e-18 | -1.96676e-17 |
| H5 | 9.27943e-17 | -3.49248e-17 | 3.72871e-17 | 5.21633e-17 | -1.97699e-17 | 1.8527e-16 | 3.19164e-17 | 4.39883e-17 |
| H6 | 8.33121e-17 | 3.19405e-18 | 4.56151e-17 | 4.6975e-17 | -5.95492e-18 | 9.80807e-17 | 8.76386e-17 | 9.89364e-18 |

SDNN

|  | 15323 | 32093 | 32182 | 40109 | 93961 | 93373 | 83782 | 83813 |
| --- | --- | --- | --- | --- | --- | --- | --- | --- |
| H0 | 7.31187 | 10.0468 | 13.7273 | 6.29114 | 10.2037 | 5.58557 | 3.92485 | 8.84781 |
| H1 | 7.80171 | 9.95921 | 12.1568 | 12.8871 | 7.21994 | 6.92882 | 5.16779 | 14.6089 |
| H2 | 8.91989 | 19.3388 | 16.6446 | 11.0034 | 17.6903 | 6.67875 | 8.04429 | 19.3063 |
| H3 | 10.4499 | 16.3619 | 24.6972 | 13.1824 | 21.5225 | 10.0713 | 41.6073 | 15.088 |
| H4 | 9.89078 | 19.8175 | 11.2847 | 14.2268 | 16.5325 | 7.2959 | 16.8454 | 19.2273 |
| H5 | 8.8375 | 21.2758 | 10.6243 | 14.6967 | 19.142 | 3.33115 | 8.95955 | 10.61 |

Evolution of heart rate variability measures in LPS group for each fetus

*Blue Singleton (n=3); Green Twin (n=5) at baseline (H0), H1, H2, H3, H4, H5, H6 after LPS injection*

*SDNN = standard deviation of normal to normal, DFA = detrended fluctuation analysis, SD2 = Standard Deviation 2, LTV = long term variability, ApEn = Approximate Entropy*
