# Supplementary material for: Early heart rate variability changes during acute fetal inflammatory response syndrome: An experimental study in a fetal sheep model
Source: PLoS One. 2023 Nov 30;18(11):e0293926. doi: 10.1371/journal.pone.0293926 (PMC10688759; doi:10.1371/journal.pone.0293926)
Supplement: Attachment — Submitted filename: response to reviewers.docx [file pone.0293926.s004.docx]

**Geoffroy Chevalier**

Department of Obstetrics

Jeanne de Flandre Hospital, CHU Lille

Avenue Eugène Avinée, 59120 Loos, France

geoffroy.chevalier@chu-lille.fr

**Editorial Office**

“PLOS ONE

**Original article entitled**

“**Early heart rate variability changes during acute fetal inflammatory response syndrome: an experimental study in a fetal sheep model**”

Dear Editor,

We would like to thank you for giving us the opportunity to submit a corrected version of our work in your journal.

We also thank the reviewers for the time spent judging our manuscript and the quality of their comments. We have responded to each of the comments and believe that the quality of the manuscript is improved by the suggestions made to us.

Corrections appear in highlighted in the manuscript and are detailed below.

We hope this revised article deserves publication in your journal,

Best regards,

On the behalf of the co-authors,

Doctor Geoffroy Chevalier, MD, PhD Student

**Reviewer’s comments:**

**“you should provide a discussion of the FHR and the large discrepancy in the timing of activation of various parameters such as SDNN. If the response is due to sympathetic nerves, I have the impression that the FHR should increase with the activation of SDNN.” “we request that the results of this peer review (1) discussion of inconsistencies in the timing of activation of various parameters”.**

It is indeed unexpected that HRV changes normalize at the same time as tachycardia appears. It has already been shown that initial increase in HRV can be followed by suppression of time-domain measures [PMID 35110628, 24944248, 18456675]. However, we agreed that with the onset of tachycardia, we might have expected an increase of indices usually associated with sympathetic activity including SDNN. We have no pathophysiological explanation for this discrepancy.

We have added this following paragraph (l363-368):

*“It is also unexpected that the onset of tachycardia from H5 was associated with a return to baseline of HRV markers.* *It has already been shown that initial increase in HRV can be followed by suppression of time-domain measures [27, 28, 29, 31]. However, with the onset of tachycardia, we might have expected an increase of indices usually associated with sympathetic activity.* *We have no pathophysiological explanation for this discrepancy”.*

**“it should be further emphasized that this model is a "non-shockable inflammation exposure model. Describing such a model as a "sepsis model" may be misleading to the reader.”**

We agree that our model is not a sepsis model. In contrast to Durosier et al (PMID: 26290042), who used exactly the same model, we have chosen to not describe it as “sepsis”. We describe the model as a “fetal inflammatory response syndrome” as described by Gomez et al (PMID 9704787, 17762416) by an elevation of fetal interleukin 6. We described it in introduction (l39) and in discussion (l315). In material and methods (l104) and in discussion (l318) we have specified that our FIRS model was “without shock or cardiovascular decompensation”.

To avoid misleading we add in abstract (l29-30): *“In our FIRS model without shock or cardiovascular decompensation”*

**“The absence of effects of cholinergic anti-inflammatory pathways in this study may be due to the fact that such inflammation was mild. The discussion section is poorly written in this revision. A comparison with a study of acute exacerbated inflammation (PMID 35110628) would add depth to the discussion.” “we request that the results of this peer review (2) comparison with changes in parameters in severe inflammatory exposure to the fetus”**

We agree that absence of effects of cholinergic anti-inflammatory pathways may be due to the fact that such inflammation was mild.

We have added this paragraph (l353-359): *We can hypothesize that cholinergic anti-inflammatory pathway was not activated because of insufficiently strong inflammation**. Indeed, Magawa et al induced acute on chronic inflammation on preterm fetal sheep with LPS bolus injections starting 48 h after LPS IV infusion.* *After a first LPS bolus, there was stronger hemodynamic response to inflammation with hypotension and tachycardia associated with initial increase in most FHRV measures, including RMSSD and HF, consistent with an activation of cholinergic anti-inflammatory pathway [PMID 35110628].*

We have also added this sentence (l406): *This experiment was not without limitations. First, it focused specifically on acute onset FIRS in the near-term fetus and the model does not therefore reflect the entire range of fetal infections, which may be subacute or chronic with differing intensity.*
