# Supplementary material for: Early heart rate variability changes during acute fetal inflammatory response syndrome: An experimental study in a fetal sheep model
Source: PLoS One. 2023 Nov 30;18(11):e0293926. doi: 10.1371/journal.pone.0293926 (PMC10688759; doi:10.1371/journal.pone.0293926)
Supplement: Attachment — Submitted filename: response to reviewers.docx [file pone.0293926.s005.docx]

**Geoffroy Chevalier**

Department of Obstetrics

Jeanne de Flandre Hospital, CHU Lille

Avenue Eugène Avinée, 59120 Loos, France

geoffroy.chevalier@chu-lille.fr

**Editorial Office**

“PLOS ONE

**Original article entitled**

“**Early heart rate variability changes during acute fetal inflammatory response syndrome: an experimental study in a fetal sheep model**”

Dear Editor,

We would like to thank you for giving us the opportunity to submit a corrected version of our work in your journal.

We also thank the reviewers for the time spent judging our manuscript and the quality of their comments. We have responded to the two comments.

Corrections appear in highlighted in the manuscript and are detailed below.

We hope this revised article deserves publication in your journal,

Best regards,

On the behalf of the co-authors,

Doctor Geoffroy Chevalier, MD, PhD Student

**Reviewer’s comments:**

**“The timing discrepancy between the occurrence of tachycardia and changes in heart rate variability parameters requires further consideration.
Please review the response time lag between these blood pressure and heart rate changes and HRV parameters in the past literature and recognize how much this study deviates from the previous study. Possible causes should then be described. Because if this is due to noise generated at a particular time, it would undermine the interpretation of the study results.”**

We agree that the timing discrepancy between the occurrence of tachycardia and changes in HRV parameters requires further discussion:

Concerning the significance of heart rate and blood pressure changes:

We used the same model of FIRS as Durosier et al (1). In their study, fetal heat rate (FHR) was significantly higher in LPS group at H6 in comparison to control group. Whereas, no significative change was found at H0, H1 and H3. We observed similar hemodynamic changes: FHR was significantly higher at H5 (196 bpm [182; 209], p = 0.01) and H6 (203 bpm [187; 223], p = 0.01) compared with baseline (173 bpm [170; 178]). In comparison to control group, FHR was significantly higher at H6.

Concerning mean arterial pressure (MAP), our results differ slightly from those of Durosier et al. In Durosier et al study, no significative change was found in MAP in comparison to control group. In our study, MAP was significantly lower only at H6 in LPS group (41.0 mmHg [40.0; 42.2]) compared with control group (47.0 mmHg [43.0; 55.5], p = 0.04). However MAP was not significantly decrease at H6 (41.0 mmHg [40.0; 42.2], p = 0.07) compared with baseline (45.5 mmHg [43.7; 48.5]).

Concerning discrepancy between the occurrence of tachycardia and changes in heart rate variability parameters:

It has been shown that HRV increase can be concomitant with tachycardia (2–4). In these studies, fetuses were premature, and fetal reactions were stronger with strong hypotension.

However, discrepancy between the occurrence of tachycardia and changes in HRV parameters has already been reported in fetal sheep at term after LPS injection (1,5). Kyozuka has also reported changes in HRV parameters in fetal sheep near term with no tachycardia (5). In their model LPS was infused into the amniotic cavity for 2 days following the 4th postoperative day to develop histological chorioamniotis. There were no significant differences in FHR after LPS injection until intra uterine fetal death. Only one HRV parameter was studied (STV: Short term variability quantifying the difference between 2 consecutive beats). This HRV parameter was significantly increase at 6, 4, and 3 h before intra uterine fetal death with no tachycardia.

Durosier el al measured 44 fHRV continuously every 5 min using continuous individualized multi-organ variability analysis (CIMVA) (1). CIMVA created a fetal HRV measures matrix across five signal-analytical domains, thus describing complementary properties of fetal HRV. Using principal component analysis (PCA), they derived and quantitatively compared the CIMVA fetal heart rate variability PCA signatures of inflammatory response in LPS and control groups.

Qualitatively, the discrimination between LPS-injected animals and control animals occurs between 2 h and 3 h post injection. Whereas FHR was significantly higher in LPS group only at H6, in comparison to control group.

It should be noted that the difference in HRV between the 2 groups persisted with the onset of tachycardia, which is not the case in our study. This may be explained by the greater sensitivity of the combined use of HRV markers compared with markers evaluated separately. Nevertheless, our results offer hope that use of HRV markers could allow detection of FIRS before the onset of tachycardia. Our results are therefore consistent with those in the literature.

We have highlighted an overall increase in global variability. As discussed in previous reviewing, inhibition or activation of autonomic activities cannot be clearly established by our HRV indices changes. We have no clear pathophysiological explanation for the discrepancy between the occurrence of tachycardia and changes in heart rate variability parameters.

We add: (l338-348)

“It has been shown that HRV increase can be concomitant with tachycardia (2–4). In these studies, fetuses were premature, and fetal reactions were stronger with strong hypotension. However, discrepancy between the occurrence of tachycardia and changes in HRV parameters has already been reported in two studies in fetal sheep at term after LPS injection (1,5). First, Durosier et al found that discrimination between LPS-injected animals and control group using HRV occurs between 2 h and 3 h post injection. Whereas FHR was significantly higher in LPS group only at H6. Second, Kyozuka et al., who studied short-term variability changes after LPS infusion into the amniotic cavity, found significative change in short-term variability, with no tachycardia, at 6, 4, and 3 h before intrauterine fetal death (5).”

**“please pay up-to-date attention to typos and misstatements”**

we have proofread the entire manuscript.

We change:

*LTV = ~~short~~ long-term variability, (l260, l546 and in S. table 2)*

1.	Durosier LD, Herry CL, Cortes M, Cao M, Burns P, Desrochers A, et al. Does heart rate variability reflect the systemic inflammatory response in a fetal sheep model of lipopolysaccharide-induced sepsis? Physiol Meas. oct 2015;36(10):2089‑102.

2.	Lear CA, Davidson JO, Booth LC, Wassink G, Galinsky R, Drury PP, et al. Biphasic changes in fetal heart rate variability in preterm fetal sheep developing hypotension after acute on chronic lipopolysaccharide exposure. Am J Physiol Regul Integr Comp Physiol. 15 août 2014;307(4):R387-395.

3.	Magawa S, Lear CA, Beacom MJ, King VJ, Kasai M, Galinsky R, et al. Fetal heart rate variability is a biomarker of rapid but not progressive exacerbation of inflammation in preterm fetal sheep. Sci Rep. 2 févr 2022;12:1771.

4.	Blad S, Welin AK, Kjellmer I, Rosén KG, Mallard C. ECG and heart rate variability changes in preterm and near-term fetal lamb following LPS exposure. Reprod Sci Thousand Oaks Calif. juill 2008;15(6):572‑83.

5.	Kyozuka H, Yasuda S, Hiraiwa T, Nomura Y, Fujimori K. The change of fetal heart rate short-term variability during the course of histological chorioamnionitis in fetal sheep. Eur J Obstet Gynecol Reprod Biol. sept 2018;228:32‑7.
